# SARS-CoV2 and Anti-COVID-19 mRNA Vaccines: Is There a Plausible Mechanistic Link with Cancer?

**DOI:** 10.3390/cancers17233867

**Published:** 2025-12-02

**Authors:** Ciro Isidoro

**Affiliations:** Department of Health Science, University of Piemonte Orientale, Via P. Solaroli 17, 28100 Novara, Italy; ciro.isidoro@med.uniupo.it; Tel.: +39-0321660507

**Keywords:** COVID-19, mRNA vaccine, lipid nanoparticle, autophagy, tumor dormancy, epigenetics, tumor microenvironment, cytokines, tumor suppressors

## Abstract

To contrast the COVID-19 pandemic brought by the corona virus SARS-CoV-2, two mRNA-based anti-COVID-19 vaccines (by Pfizer-BioNTech and Moderna) were made available relatively quickly and deployed worldwide based on an emergency approval. Being considered vulnerable and at risk of infection, cancer patients have been prioritized for COVID-19 vaccination and vaccinated repeatedly because of the short time protection provided by these vaccines. Recently, a surge in the incidence and rapid progression of cancers has been observed in many countries, which could (at least partially) represent cancers undiagnosed or untreated during the pandemic. It has also been suggested that the SARS-CoV-2 itself or even the anti-COVID-19 mRNA vaccines could have contributed to the recurrence and worse clinical outcome in cancer patients, given the high incidence of COVID-19 in hospitalized patients and that these patients have been vaccinated with priority several times and in a short period. Although it appears extremely unlikely that SARS-CoV-2 and anti-COVID-19 mRNA vaccines elicit genotoxic events and cause neo-cancerogenesis in a short time, they could still cause non-genotoxic pro-carcinogenic effects by triggering an exaggerated inflammatory reaction, compromising immune homeostasis, stimulating cell proliferation, and negatively affecting cellular stress response and damage repair machinery. This could result in the promotion of regrowth of dormant micrometastases or relapses of stable minimal residual disease. Such a harmful outcome may likely result from a synergy between the virus and the vaccine, especially in multi-vaccinated and multi-infected individuals. Here, I bring the cell pathologist’s point of view and discuss the multiple possible mechanisms by which the virus and the anti-COVID-19 mRNA vaccine might favor tumorigenesis. While a causal link cannot be established at this stage, knowledge of potential carcinogenic risks could help doctors and health policymakers take the best actions to protect vulnerable patients and convince the vaccine developer to design a vaccine free from such harm.

## Simple Summary

Between 2021 and 2024, large segments of the population—including cancer patients—experienced multiple COVID-19 infections and received repeated doses of mRNA vaccines. The concomitant increase in newly diagnosed cancers or fast progression of cancers under treatment raised concerns about whether SARS-CoV-2 or the vaccines might play a role in these outcomes. While it appears extremely unlikely that SARS-CoV-2 and anti-COVID-19 mRNA vaccines can elicit genotoxic events causing neo-cancerogenesis in a short time, they could still cause non-genotoxic pro-carcinogenic effects. Indeed, multiple molecular, cellular, and systemic mechanisms, including disruption of the immunosurveillance and induction of inflammation in the tumor microenvironment, disruption of autophagy and of tumor suppressor pathways, and activation of signaling involved in cell proliferation and cell migration, could synergistically lead to the awakening and fast growth of dormant microtumors, particularly in vulnerable individuals exposed to both repeated infections and vaccinations over a short time frame.

## 1. Introduction

In March 2020, the WHO officially declared COVID-19, the infection brought by the coronavirus SARS-CoV2, as a global pandemic. At that time, because of the large number of infected patients requiring assistance, many hospitals opted to give priority to these patients, converting specialist wards into COVID-dedicated wards, and this has inevitably postponed specialist treatment for other pathologies, including cancer [1,2]. As a result, diagnosis and medical care at earlier stages of disease were negated to a large population [3].

A few months later (i.e., December 2020 and January 2021), the administration of mRNA-based anti-COVID-19 vaccines (BNT162b2/Comirnaty from Pfizer-BioNTech and mRNA-1273/Spikevax from Moderna), manufactured with a novel technology and approved for emergency use, was prioritized for elders (over 60 s) and “vulnerable” (so-called “frail”) patients with chronic diseases such as neuro-degenerative disorders, autoimmune diseases, and cancer particularly among others [4,5]. Soon after, vaccination was made mandatory for health care workers and thereafter for the general population to be able to work in public settings. As of August 2023, a total of 84.8% of the European Union adult population had been vaccinated at least once against the virus (https://commission.europa.eu/strategy-and-policy/coronavirus-response/safe-covid-19-vaccines-europeans_en; accessed on 31 August 2025). As of April 2023, almost 400 million doses of Pfizer-BioNTech and 250 million doses of Moderna vaccines have been administered in the United States (https://www.statista.com/statistics/1198516/covid-19-vaccinations-administered-us-by-company/; accessed on 31 August 2025). Since then, the rate of vaccination has steadily declined in all Western countries.

During the pandemic, almost 775,615,736 confirmed cases and over 7,051,323 deaths have been attributed to COVID-19 by the World Health Organization (WHO) (https://covid19.who.int/; accessed on 28 June 2024). According to a recent study, during the period of December 2020 through March 2023, the anti-COVID-19 vaccines would have saved up to 1.6 million of lives among people aged >25 years old in European countries [6].

The COVID-19 pandemic was officially declared terminated on 5 May 2023 [7], yet WHO warns the governments on the need to continue the anti-COVID-19 vaccination campaign as a preventive measure to reduce the hospitalization burden. Again, cancer patients are forced to vaccinate against COVID-19 as per the recommendation of the scientific societies of oncologists (ASCO, American Society of Clinical Oncology in US, and ESMO, European Society of Medical Oncology in Europe, and others similar). Comirnaty (Pfizer-BioNTech) and Spikevax (Moderna) remain the most widely used anti-COVID-19 vaccines.

Presently, the worst of the virus seems to be over. On the opposite, cancer is on the rise worldwide [8], with almost 20 million new cases and 9.7 million cancer-related deaths in 2022, and it is predicted to further increase to up to 35 million new cases in 2050 (https://www.uicc.org/news/globocan-2022-latest-global-cancer-data-shows-rising-incidence-and-stark-inequities; accessed on 30 December 2024). In the United States, the cancer mortality rate decreased by 33% from 1991 through 2021 [9]. In 2025, it is estimated that two million new cases of cancer will be diagnosed and about 618,000 people will die from cancer in the United States (https://www.cancer.gov/about-cancer/understanding/statistics; accessed on 31 August 2025). A recent study based on a mathematical model predicting cancer incidence and mortality rates in Australia indicates that the number of new diagnoses will increase by 51% and mortality will increase by 36% between 2020 and 2044 [10].

More worrisome is the recent projection showing an increased incidence of cancer at a younger age (<50) for the generation born in 1965–1980 compared to the generation born before 1964 [11]. The style of life is blamed as the main culprit for such trend, though other factors, including infections and medications that negatively impact on the immune system and metabolic homeostasis, should not be neglected.

The case reports describing the sudden occurrence of rapidly progressing cancers diagnosed at an advanced stage in otherwise healthy patients or the relapse and fast progression of cancers in cancer-bearing patients after anti-COVID-19 vaccination are increasing in the peer-reviewed literature, not considering the retracted one (see Table 1 and Table 2). Consequential and time correlations, which do not imply a causal correlation per se between the vaccination campaign and such increased incidence of cancer, have raised concerns about the possible causal link. However, establishing a causal link is challenging because the national cancer registries do not consider the newly diagnosed or the recurrent cancers as possibly linked to the vaccination status. On the other hand, prospective active pharmacovigilance that matches vaccinated and unvaccinated individuals, healthy or cancer carriers, has not been pursued in the last three years, and would in any case be impracticable because both populations of healthy individuals aged >60 years (those more susceptible to developing cancer) and cancer patients have largely been vaccinated. Adding to the complexity, these patients likely have also been infected with SARS-CoV2 before and/or after vaccination. Now that five years have passed since the spread of the virus throughout the world and that the vaccine has been administered to a large population for three years, we can take stock and try to answer the fundamental questions: (i) Is it plausible that the COVID-19 virus and the anti-COVID-19 mRNA vaccines may cause cancer? And, if yes, (ii) how much have they contributed and how much will they contribute in the future to the increase in cancer? When addressing these questions, we must consider that cancer intrinsically tends to worsen (despite the treatment), and that these patients have been vaccinated three or more times and, most likely, have also contracted COVID-19 [12,13,14]. Thus, it is objectively difficult to determine and weigh the factor(s) causing clinical worsening in cancer patients. Similarly, in the case of (apparently) healthy individuals who experience the sudden onset of cancer after vaccination, we must consider possible previous SARS-CoV2 infections or other predisposing factors that could have favored carcinogenesis. In the latter case, the virus and the vaccine could still have had an add-on triggering role. But one thing must be clarified immediately: the concept of “turbocancer” developing in little more than two years has no scientific basis, even in the case of injecting carcinogenic chemicals into the bloodstream.

Here, I will not delve into the causality assessment, which would be intricate and challenging [15], and instead will present and discuss the potential mechanisms and pathways through which the SARS-CoV2 virus and the anti-COVID-19 genetic vaccines could contribute to carcinogenesis or worsening of pre-existing tumors. This knowledge is useful for informing policymakers and clinicians in choosing the best public health intervention to protect citizens and patients facing similar viral pandemics in the future. The objective is not to blame cancer on the virus or anti-COVID-19 vaccines but rather to instill doubts and stimulate reflection free from any prejudice, dogmas, and conflict of interest on the safety of these mRNA vaccines and on the best precautions to implement to protect patients at risk of viral infections.

## 2. The Virus, the Cancer, and the mRNA Vaccine: The Ugly, the Bad, and the Good?

Parodying the cult film “*The Good, the Bad, and the Ugly*”, we can say for sure that the “Ugly” is the virus and the “Bad” is the cancer, but are we sure that the vaccine is the “Good”?

### 2.1. The Cancer

Let us start with the “Bad” guy. Cancer is not a single well-defined disease, rather it is a complex multifaceted disease that evolves continuously and dynamically changes its features in response to local and systemic environmental signals [16,17]. At the time of cancer diagnosis, we face a mass that is constituted by many different malignant clones of cells that behave differently (in terms of proliferation, metabolism, survival, migration, and other characteristics) and that very likely have already spread in other body districts to form metastases, some of which may not be detectable by diagnostic imaging [18]. During this process, other cells in the same tissue may undergo transformation into cancer cells and start that very same process of clonal evolution and spreading. Thus, when the patient eventually calls upon a doctor because of the symptoms, the body is likely to have many different cancers at various degrees of progression in one or more organ(s). According to the “somatic mutation theory”, transformation into a cancer cell results from the accumulation of mutations in the functioning of several genes (belonging to the families of oncogenes, tumor suppressor genes, and DNA repair genes) that control cell proliferation, cell differentiation, cell death, cell migration, cell metabolism, and systems for repairing the DNA and protein/organelle damages [18]. Mutated functioning of these genes results from either genetic change in their DNA coding sequences or from epigenetic changes in their expression [19,20]. Unrepaired genetic and epigenetic mutations in so many genes accumulate over many years, and this explains why spontaneous (sporadic) cancers develop in decades and are in fact diagnosed more frequently in the 70s [9]. However, this can be anticipated in cases of chronic exposure to environmental mutagenic/epimutagenic factors (so-called “genotoxic and non-genotoxic carcinogens”) and/or malfunctioning (in some cases genetically inherited) of the machineries that keep under check the cellular damages and the abnormal cell behavior [21,22]. However, in some cancers, massive genomic alterations have been shown to occur as a single catastrophic event in a short time [23].

Still, the presence of mutated oncogenes and tumor suppressor genes is not sufficient for the development of cancer because the surrounding microenvironment can build a barrier that contrasts the proliferation and spread of such transformed cells [24,25]. Even more intriguingly, tumors without genetic alterations have also been described, which calls into question the “somatic mutation theory” [26].

### 2.2. The Virus

What can we say about the “Ugly” guy? SARS-CoV2 was so named due to its high similarity with SARS-CoV, the coronavirus that caused the very similar respiratory distress syndrome described by Carlo Urbani in 2003 [27]. COVID-19 may present with mild to severe flu-like symptoms, though in certain patients, particularly elders and those with co-morbidities (such as cardiovascular, diabetes, obesity), the disease can rapidly progress and lead to death following respiratory distress and multi-organ failure arising from a hyperactivation of the inflammatory response associated with hyperproduction of cytokines and multiple thromboembolisms [28,29]. The spheric virion (approx. 100 nm in diameter) consists of an envelope made of a by-layer lipid membrane inserted with the structural proteins E (Envelope), M (Membrane), and S (Spike, a highly glycosylated protein that assembles as trimers) and containing a 29.9 kb single-stranded, positive-sense RNA filament complexed with the Nucleocapsid protein (N) [30]. The virus exploits the Spike (S) protein to infect the cells through binding to the angiotensin-converting enzyme 2 (ACE2) protein expressed on the membrane of endothelial and epithelial cells of various organs, particularly the lungs, intestine, and kidneys [30,31]. The SARS-CoV2 Spike protein presents the unique polybasic sequence (681PRRAR685) for the furin-mediated cleavage into the two subunits S1 (aa 1–685, that contains the ACE2 binding domain) and S2 (aa 686–1273, that mediates the virion envelope fusion with host cellular membrane), and this peculiar feature is believed to enhance the virus cellular infectivity and transmissibility [25]. Virus entry is also facilitated by the proteolytic cleavage of the S protein (at the furin site) by the recipient cell-surface serine protease TMPRSS2, which promotes the virion-cell fusion mediated by the S2 subunit.

Endocytosis and endosomal cysteine proteases cathepsins B and L can also contribute to the virus entry and thereafter release of the viral RNA in the cytoplasm. Once entering the cell, the viral RNA is freed in the cytoplasm and is copied as full length, for inclusion in the new virions, and as sub-genomic RNA fragments for directing the synthesis of the structural and accessory proteins. The whole genome codes for the four structural proteins (E, M, S, and N), two polyproteins (ORF1a and ORF1b), and six accessory proteins (of unknown function) [30,31]. The polyproteins ORF1a and ORF1b are proteolyzed, respectively, by the Papain-like protease (PLpro = nsp3) and the 3-chymotrypsin-like protease (3CLpro, aka Main protease Mpro = nsp5) to generate 16 nonstructural proteins (NSP 1–11 and NSP 12–16, respectively) necessary for viral replication and assembly [30,31]. Virus replication involves the formation of endoplasmic reticulum-derived double-membrane vesicles that share similarities with autophagosomes, and the assembled virions then leave the cell by exocytosis passing through the Golgi complex or the secretory lysosome pathway [32,33]. The autophagy–lysosomal pathway plays a dual role in viral infection and replication: on the one hand, it can lead to lysosomal degradation of the whole virion, yet on the opposite, it can be manipulated by the virus to serve as the membrane platform (the double membrane vesicles) for its replication and assembly [33,34]. SARS-CoV2 can in fact be directed for lysosomal degradation once it enters via endocytosis or it is in the cytoplasm, yet certain viral proteins (namely NSP6) can impair the autophagosome–lysosome fusion and lysosomal degradation of the viral particles resulting in the accumulation of autophagosomes [33,35]. Autophagy also plays a role in innate and adaptive (MHC-dependent antigen presentation) immune responses [36]. Therefore, SARS-CoV2 disruption of autophagy in antigen-presenting cells can compromise the anti-viral immune response. Since autophagy opposes to inflammasome biogenesis in immune and epithelial cells, the disruption of autophagy by SARS-CoV2 may lead to inflammasome-dependent pyroptosis in infected airway cells [37] and cytokine storm and thromboembolisms [38,39]. The question here is whether cancer patients are more likely to be infected by SARS-CoV2 and, in that case, what could be the biological explanation. It is a fact that COVID-19 is diagnosed with high frequency (and often with fatal outcome) in cancer patients, and the simplest explanations are the hospitalization (where the probability of encountering the virus is high) and immunodepression arising from the cancer itself and the treatments [40]. Yet, it has been argued that, paradoxically, immunodepression could protect SARS-CoV2-infected cancer patients from the fatal risk of hyper-cytokinemia [41].

As for factors that may favor infectivity in cancer patients, the most obvious ones to consider are the cell surface ACE2 (the SARS-CoV2 receptor) and TMPRSS2 (the enzyme that processes the Spike protein to facilitate the cellular entry of SARS-CoV2). High levels of ACE2 and of TMPRSS2 were found, respectively, in renal, colorectal, and gastric carcinomas and in prostate, colorectal, and esophagogastric carcinomas [42]. It has been hypothesized that patients bearing such cancers are more prone to be infected and to exhibit the severe form of COVID-19. In prostate cancer cells, TMPRSS2 has been shown to activate protease-activated receptor-2, triggering downstream signaling pathways associated with inflammation, metastasis, and invasion (see below). Interestingly, androgen deprivation therapy decreases the expression of TMPRSS2 and reduces the risk of SARS-CoV2 infection in prostate cancer patients [43]. In addition to ACE2, other membrane proteins may act as Spike receptors (or co-receptors) for SARS-CoV2 among which we only mention AXL [44], Neuropilin-1 [45], and CD147 [46] that are highly expressed on cancer cells and might explain the increased susceptibility of cancer patients to infection [47,48,49].

There is also the possibility that susceptibility to SARS-CoV2 infection and to cancer development shares some genetic and epigenetic factors. Genomic and epigenomic studies have delineated the host genetic determinants of COVID-19 susceptibility and of the clinical outcome [50,51,52,53]. In a study correlating gene and protein expression of 17 COVID-19 susceptibility genes with lung cancer prognosis, it was found that the hyperexpression of FYCO1, CXCR6, XCR1, and TAC4 in cancer cells was protective, whereas that of TMEM65 and OAS1 was a risk factor for SARS-CoV2 infection [54]. Another study found that the genetic predisposition to colorectal or lung cancer was causally associated with a decreased or increased susceptibility to COVID-19 severity, respectively, and this association pointed to the LZTFL1, CCR9, FYCO1, CXCR6, XCR1, and ABO genes [55]. Notably, gene mutations altering the tertiary structure of the FYCO1 protein were associated with increased viral replication and spread via enhanced exocytosis, which could explain the severity of COVID-19 [56].

### 2.3. The Vaccine

Finally, in 2021, what was hailed as the “Good” guy, i.e., the vaccine, entered the scene. The two mostly used anti-COVID-19 mRNA vaccines, manufactured by Pfizer-BioNTech and Moderna, have been approved under emergency circumstances by drug regulatory agencies (FDA in US and EMA in EU) for the prevention of COVID-19 disease in individuals 16 years of age and older. Approval was based on a 3-month trial demonstrating greater than 94% (relative) efficacy in preventing infection and severity of outcomes and showing only mild-to-moderate reactions in the 2 months after the second dose [57,58]. It is to be stressed that cancer patients were not included in these clinical trials.

Both these vaccines are made of lipid nanoparticles (LNPs) containing the coding mRNA for the Spike protein. In this sense, they do not act like traditional protein-based vaccines in that the immunogenic protein is synthesized by the host, which makes this product more like an “immunomodulatory genetic pro-drug” (for the sake of simplicity, hereafter I will call it “mRNA pro-vaccine”). It has also been noticed that these “mRNA pro-vaccines” do not contrast SARS-CoV2 infection, instead they induce the synthesis of neutralizing IgG that can limit the reproduction and organ spread of the virus, thus attenuating the clinical symptoms of the disease [59], and for this, they are better known as anti-COVID-19 vaccines. This is also due to the inability of intramuscular injection of this mRNA pro-vaccine to trigger the production of anti-Spike mucosal IgA even after three doses [60].

An exaggerated, yet transient, immune-inflammatory response at the axillary lymph nodes, occasionally associated with alterations in the ipsilateral parenchyma, following anti-COVID-19 mRNA vaccination, is a quite frequent finding and might raise the suspicion for malignancy [61,62].

Of more concern is the fact that the multiple vaccinations with these products shift the immune response to a tolerant response where the inert subclass IgG4 are predominantly produced [63], particularly in patients that are infected after the vaccination [64]. The question here is “How much has it revealed to be useful and safe for cancer patients to be vaccinated for COVID-19 with these mRNA pro-vaccines?”. Cancer patients are generally immunosuppressed, as a side effect of both the treatments (many chemotherapeutics are myelosuppressive) and the disease itself, and this makes them more vulnerable to infections, and hospitalization itself increases the risk of exposure to bacteria and viruses. Not surprisingly, patients with solid or hematologic cancers, and particularly those with metastases, were shown to be more susceptible to contract the severe form of COVID-19 [65,66].

Anti-influenza (traditionally made) vaccines are routinely administered to onco-hematologic and solid cancer-bearing patients, based on the assumption that immunization will protect these vulnerable patients from severe outcomes with no side effects. Based on these considerations and on the results of the clinical trials claiming a relative (not absolute) 95% efficacy [57,58], the anti-COVID vaccination has been prioritized for cancer patients [65,67], neglecting the fact that those clinical trials did not include such a typology of patients [57,58]. Several studies have shown that influenza vaccination fails to elicit the expected protection in patients with solid cancer or hematological malignancies [68,69]. It has also been emphasized that cancer patients should not be vaccinated while under radio- or chemotherapy because of the inefficacious immune response [70]. ESMO’s press release on 20 Sept 2021 emphasized the data reported at the ESMO Congress 2021 proving the safety and the protective efficacy of two or, better, three vaccine doses in cancer patients (https://ecancer.org/en/news/20966-esmo-2021-evidence-suggests-that-covid-vaccines-do-protect-patients-with-cancer; accessed on 27 June 2024). A multicenter study in a cohort of 84 non-vaccinated and 49 vaccinated (majority with mRNA-based vaccine; one-third with three doses) cancer patients who tested positive for SARS-CoV2 reported that in the latter group, COVID-19 was milder and the vaccine better protected from COVID-19-related death [71]. These reports were based on a relatively short period of observation and from a few cohorts. A few months later (in June 2022), the first real-world data analysis showed that breakthrough infections, even with severe outcomes, may occur in cancer patients vaccinated with mRNA-based anti-COVID-19 vaccines (the rate was lower for Moderna vaccine compared to the Pfizer vaccine) [72]. Compared with healthy controls, cancer patients receiving three doses of the Comirnaty (Pfizer-BioNTech) mRNA vaccine showed a lower cell-mediated immune response and lower anti-Spike antibody titers, indicating the need for additional boosters to provide protection [73,74].

Other studies confirmed that vaccinated cancer patients can contract the SARS-CoV2 infection and that those under treatment, particularly the hematologic patients receiving anti-CD20 therapy, have an increased risk for severe COVID-19 [12,14,75,76].

Patients bearing solid cancers under treatment showed sub-optimal seroconversion in response to anti-COVID-19 mRNA pro-vaccine [77] and may develop serious immune-related adverse effects [78]. Compared with matched healthy patients, the humoral immune response to BNT162b2 (Pfizer-BioNTech Comirnaty) mRNA pro-vaccine was markedly lower in B chronic lymphocytic leukemia patients under treatment with Bruton’s Tyrosine Kinase inhibitors or venetoclax ± anti-CD20 [79].

Corticosteroids are routinely administered to cancer patients as co-medication and, given their immune-suppressive activity, one may expect a low vaccination efficacy in these patients [80]. Similarly, since PD-1 blockade impairs the CD8 response to antigenic stimulation [81], it is likely that therapies with immune-checkpoint inhibitors in solid cancer patients would abrogate the T-cell specific response to COVID-19 vaccination. While chemotherapy has been reported to interfere with seroconversion, immunotherapy appears to not compromise the humoral response to mRNA-anti-COVID-19 vaccination in cancer patients; yet in these patients, the production of autoantibodies has been reported, raising concerns about the risk of developing autoimmune diseases [82]. Autoimmune-related adverse effects have been reported after the third dose of COVID-19 mRNA pro-vaccine (Comirnaty) in cancer patients under treatment with immune-checkpoint inhibitors [83]. These observations caution that introducing mRNA-based immunostimulants along with immunosuppressive therapies in the context of a dysregulated immune system (such as in cancer patients) may have unpredictable consequences. The mRNA anti-COVID-19 vaccination exacerbated the pro-inflammatory Th17 immune response along with neutrophilia in oncologic patients, particularly in those recovered from COVID-19 [84]. Since this condition poses the risk of triggering cytokine storm, it requires caution in vaccinating cancer patients with previous SARS-CoV2 infection, and this is of particular concern when multiple boosters are administered. A recent literature review concluded that COVID-19 vaccination was generally well tolerated, safe, and effective in cancer patients, with rare severe side effects including necrotizing myopathy, thromboembolisms, and allergic reactions [85]. Yet, this same study also revealed that protection was moderate and limited in time, since despite the vaccination, cancer patients could get infected, with hospitalization and a high risk of mortality, requiring continuous booster doses as a preventive measure [85]. Indeed, T-cell response (which is the most important to combat viral infections) in cancer patients was weak even after the third dose [73]. In this regard, concerns have been raised about the possibility that multiple boosters could induce CD8+ T-cell exhaustion along with increased expression of PD1 [86]. In line with the above, patients with chronic lymphocytic leukemia showed an impaired CD4+ and CD8+ T-cell memory response to viral Spike eight months after two doses of the Comirnaty (BNT162b2) mRNA vaccine [87]. Overall, these studies question the assumption that COVID-19 vaccination is beneficial in terms of protection against COVID-19 infection and clinical outcomes and instead raise important concerns about its safety for cancer patients, especially because short-term protection imposes continuous booster vaccination.

## 3. The Complex Interplay Between COVID-19, Anti-COVID-19 mRNA Pro-Vaccine, and Cancer

We cannot close this first introductory section without mentioning case reports suggesting a paradoxical effect of the SARS-CoV2 infection and of the anti-COVID-19 vaccination, in combination or alone, associated with a partial and transitory regression of cancer. In a small cohort of cancer patients undergoing checkpoint immunotherapy, an increase in the absolute number of circulating NK cells, not of T and B cells, occurred four weeks after the third dose of the Comirnaty vaccine, and these patients showed a reduced likelihood of disease progression within six months of vaccination [88]. To be noted, this is the same cohort in which one-fifth of the high antibody responders to vaccination developed autoimmune thyroiditis [83].

A very recent study shows that certain patients bearing aggressive forms of skin and lung cancer who received COVID-19 mRNA vaccines within a hundred days of their immune checkpoint therapy experienced a surge in type I interferon response that enhanced T-cell response and led to improved survival by a few months [89].

In their review, Meo et al. describe the clinical cases of nine patients with hematological malignancies (including lymphomas, leukemias, and myelomas) and five patients with solid tumors (two renal tumors and three colorectal tumors) in whom a spontaneous temporary remission (the longest was up to 12 months) was observed following SARS-CoV2 infection [90]. The authors’ explanations for this effect include a possible direct oncolytic effect of the virus in infected malignant lymphocytes and the stimulation of T-cytotoxic cells by pro-inflammatory cytokines within the solid tumor microenvironment, though no mechanistic studies were performed to confirm such biological and immunological activities.

In the literature, there are also three cases of partial cancer regression following the anti-COVID-19 vaccination. In one case, a patient diagnosed with left parotid myoepithelial carcinoma and possible metastatic nodules in the lung received two doses of the Moderna mRNA-1273 COVID-19 vaccine, to which they experienced a severe adverse reaction that resolved within two weeks, and over the next 9 months, showed a 73% reduction in tumor burden associated with a phenotypic shift in the tumor immune microenvironment from a pro-tumorigenic (characterized by M2 macrophages) to a pro-inflammatory anti-tumor phenotype enriched in CD8+ T cells [91]. In a patient affected by primary cutaneous anaplastic large-cell lymphoma (pcALCL) showing recurrence and multiple lung nodules (suspicious of metastases) after therapy, a marked regression of the cervical lymph node and lung lesions was observed one week after administration of one dose of Comirnaty vaccine, suggestive of a possible causal correlation [92]. The authors, however, correctly mention that pcALCL frequently undergoes spontaneous regression. A third case refers to a patient diagnosed with cutaneous Merkel Cell Carcinoma (MCC) who experienced the regression of an enlarged axillary metastatic lymph node after the third dose of Comirnaty vaccine [93]. As noticed by the authors, despite being highly aggressive, spontaneous regression of MCC is relatively frequent. The latter two cases may be explained with the intrinsic propensity of spontaneous regression of small metastatic lesions possibly favored by vaccine-induced immune stimulation. Finally, there is a case of a patient who had recurring hepatocarcinoma four months after partial hepatectomy and six months later showed regression of the hepatic lesion after three doses of Moderna mRNA-1273 and SARS-CoV2 infection [94]. This latter case points to the complex interplay between the host immune response in cancer patients, COVID-19, and mRNA anti-COVID-19 vaccination. These few case reports remain anecdotal and present important limitations for establishing any causal correlation or generalizability, especially when considering the lack of clear mechanistic explanation, insufficient reproducibility, and the larger number of cases where such an effect has not been reported or, instead, an opposite effect has been reported, as discussed in the next paragraphs.

## 4. Can SARS-CoV2 and/or Anti-COVID-19 mRNA Pro-Vaccine Cause Cancer? Putting the Puzzle Pieces Together

In this second part, we will address the question whether and how the SARS-CoV2 and the anti-COVID-19 mRNA pro-vaccine can cause cancer or worsen the prognosis of pre-existing cancers.

We have learned that cancer is a dynamically evolving proliferative and invasive disease arising from the accumulation of genetic and epigenetic changes in the parenchymal cells whose growth and spread are facilitated by microenvironmental factors. Preneoplastic nodules, micrometastases, and residual disease (after surgical debulking and anti-cancer therapy) may remain stable for decades in a dormant state due to insufficient blood supply (angiogenic dormancy), due to efficient immune suppression (immune-mediated dormancy), and due to up-regulated autophagy (autophagy-mediated cancer cell dormancy) [95,96,97]. Tissue inflammation is the main cause of dormancy interruption and cancer outgrowth, by promoting neoangiogenesis and immune suppression, while inhibiting cellular autophagy [95,98,99,100]. The role of growth factors and hormones, neoangiogenesis, inflammation, and immune-suppressive cells in the microenvironment in the growth of metastases was outlined by Stephen Paget (1889) in his “seed and soil” theory [101]. Another important feature of cancer cells is the alteration of the glucose and amino acid metabolisms so that glucose is preferentially glycolyzed with the production of lactic acid while mitochondria preferentially utilize glutamine for the Kreb’s cycle [102].

Therefore, to induce or promote carcinogenesis, the virus and the mRNA pro-vaccine must possess one or more of the following abilities: 1. induce gene mutagenesis; 2. induce epigenetic changes; 3. interfere with the oncogenic and tumor suppressor pathways that control cell behavior and fate impinging on cell proliferation and cell migration, autophagy, cell survival and cell death, and energetic metabolism; 4. induce inflammation, angiogenesis, and lymphopenia in the tissue microenvironment.

**Figure 1:**
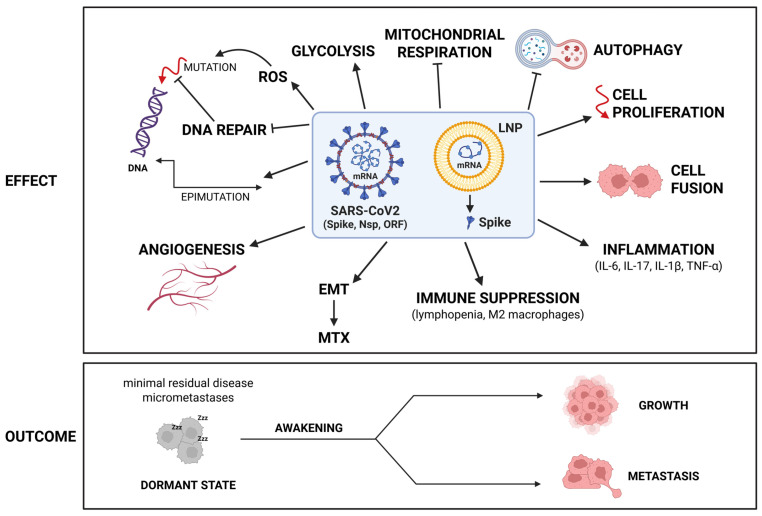
SARS-CoV2 and anti-COVID-19 mRNA pro-vaccine may trigger several pro-carcinogenic pathways while impairing anti-cancer processes. The virus and the mRNA pro-vaccine share some of these effects triggered by the Spike protein (though the vaccinal Spike is modified), while other effects are unique to the virus (triggered by viral proteins) or to the pro-vaccine (the LNP), as detailed in the following sections.

While the possibility that the viral genetic code or the reverse-transcribed pro-vaccine mRNA could integrate into the cellular genome and cause gene mutagenesis is deemed extremely unlikely, all other events have indeed been associated with SARS-CoV2 infection and anti-COVID-19 vaccination. These concepts are illustrated schematically in Figure 1.

Although some events are uniquely associated with the peculiar individual physical-chemical structure of the virus or anti-COVID-19 mRNA pro-vaccine, we will see that both share the characteristics to trigger the very same events. In particular, the viral Spike protein and that produced by the vaccine mRNA, being structurally very similar, will likely trigger the same reactions. Another major cancer-promoting mechanism shared by SARS-CoV2 infection and anti-COVID mRNA pro-vaccines is inflammation.

## 5. The SARS-CoV2 Virus and the Cancer

Recent studies pointed out the possibility that SARS-CoV2 infection might create conditions for cancer progression [103,104]. A special and obvious suspect is IL-6 due to its role in COVID-19-associated inflammation [105]. However, as we will see in detail, many other factors and pathways can link SARS-CoV2 infection with cancer, with the Spike protein as the main trigger.

### 5.1. Oncogenic Potential of SARS-CoV2 Receptors ACE2 and AXL

In SARS-CoV2-infected patients, as well as in anti-COVID-19 mRNA pro-vaccinees, membrane-bound and circulating ACE2 levels are decreased due to Spike attack, and this has been linked to inflammation, thrombosis, and hypertension [106,107,108]. Could the depletion of ACE2 have a role in carcinogenesis? ACE2 is a peptidase that can be found as membrane bound on the surface of endothelial and epithelial cells and as a soluble form in the circulation. It has a pivotal role in the Renin–Angiotensin System, which controls cardiovascular functioning. In brief, the liver secretes in the blood angiotensinogen that is processed to angiotensin I (AngI) by Renin (secreted by the kidneys), and AngI is further processed by the enzyme ACE (angiotensin converting enzyme; expressed particularly, not exclusively, in the epithelial cells of the lungs) into the vasoconstrictor AngII, which can eventually be processed by ACE2 into the vasodilator peptide Ang1-7. Thus, while AngII favors hypertension, Ang1-7 contrasts hypertension, dampens inflammation, and prevents thromboembolisms [108]. More than that, AngII has mitogenic and angiogenic activities and inhibits cancer cell apoptosis, whereas Ang1-7 inhibits angiogenesis and cancer growth [109,110]. Consistently, ACE2 was reported to inhibit angiogenesis and to prevent metastasization in breast and lung cancer models [111,112,113]. ACE2 deficiency was shown to increase the risk of hepatocarcinogenesis and the resistance to anti-PD-L1 immunotherapy, while promoting a permissive tumor microenvironment associated with M2-like macrophages, angiogenesis, and immunosuppressive myeloid cells [114]. SARS-CoV2 promoted the Epithelial-to-Mesenchymal Transition (EMT) of infected lung cancer cells, associated with high expression of ZEB1 and AXL and decreased expression of membrane ACE2 [115].

In benign mammary epithelial cells transgenically expressing ACE2, the challenge with SARS-CoV2 Spike protein induced the transcription of SNAIL and acquisition of a migratory and invasive mesenchymal phenotype [116]. Further, the hyperglycosylated Spike protein from the SARS-CoV2 gamma variant was shown to induce SNAIL-mediated EMT and to promote in vivo metastasization of xenografted human breast cancer cells [117].

Thus, depletion of ACE2 results in the loss of an anti-cancer barrier against the growth and spread of pre-existing (micro)tumors, favoring metastasization [118,119,120]. In this context, it is worth mentioning that ACE2 can be targeted by MDM2 (mouse-double-minute 2) and thereafter ubiquitinated and degraded via proteasome [121]. To be noted, MDM2 is considered an oncogenic protein since it can direct the proteasome degradation of TP53, a major tumor suppressor protein (see Section 5.3). It is tempting to speculate that Spike-induced ACE2 depletion might leave MDM2 free to bind and direct the degradation of TP53, thus further increasing malignancy (more in Section 5.3).

Another receptor for SARS-CoV2 potentially linking COVID-19 to cancer is AXL (Anexelekto). This is a transmembrane receptor protein (its physiological ligand is GAS6) that plays an important role in cancer progression, as its activation promotes cell proliferation, EMT, and metastasization [122]. It is worth noting that ACE2 and AXL are involved in other cancerogenic pathways, as will be explained in detail below.

### 5.2. SARS-CoV2 Spike Protein Can Trigger Oncogenic Signaling Pathways

Human lung carcinoma A549 cells (type II pneumocyte) incubated with SARS-CoV(1)-like particle or its isolated Spike showed the Casein Kinase II-mediate phosphorylation of ACE2 and the activation of the Ras-ERK (extracellular regulated kinase)-AP1 pathway [123]. More recently, the S1 subunit of SARS-CoV2 Spike protein was shown to trigger the ERK signaling in lung endothelial cells, and this effect was not mediated by the interaction with ACE2 [124]. In lung carcinoma A549 and in hepatocarcinoma Huh-7.5 cells, the SARS-CoV2 Spike activated the MAPK-NF-κB pathway and downstream induction of IL-6 synthesis [125]. In lung epithelial cells, SARS-CoV2 was shown to activate the Epidermal Growth Factor Receptor (EGFR)-AKT survival signaling pathway along with stimulation of mitochondrial ATP production [126]. This mechanism is believed to help the virus to sustain its replication by keeping alive and boosting the energy metabolism of the infected cells in the early phase of infection.

The (Ras-)ERK/MAPK and the AKT pathways drive transcription, protein synthesis, cell proliferation, and cell survival, and are hyperactivated in cancer cells [127,128].

Additionally, the Spike proteins were shown to interact with the Estrogen receptor and induce ERα-dependent cell proliferation of breast cancer cells [129].

Finally, an in silico study found that the viral Spike protein potentially interacts with and activates the EGFR and VEGFR pathways [130].

Whether the above signaling triggered by viral Spike could result in the aberrant survival and stimulation of proliferation and migration of infected pre-neoplastic cells has not been investigated yet, and it cannot be excluded.

### 5.3. SARS-CoV2 Spike Protein Can Inactivate Tumor Suppressor Signaling Pathways

TP53 and BRCA1/2 are two major tumor suppressor proteins that play a major role in cancer progression and therapy resistance [131,132]. TP53 (p53) has nuclear and cytosolic functions: in the nucleus, as a homo-tetramer, it binds the DNA to direct the transcription of genes that regulate the cell cycle, DNA repair, apoptosis, autophagy, and cell metabolism; in the cytoplasm, as a monomer, it directs the BAX oligomerization on the outer mitochondrial membrane and lysosome membrane for inducing cell death [133]. Certain p53 mutants unable to bind the DNA may act as “dominant negative” and impair apoptosis and autophagy [134].

A complex interplay between p53 and the SARS-CoV was previously found: p53 was shown to be able to inhibit viral replication and, on the other hand, the virus can promote the ubiquitination and degradation of p53 [135]. Given the similarity between the domains involved in SARS-CoV and SARS-CoV2, it is reasonable to hypothesize that the latter also has a similar relationship with p53.

In this context, an in silico study found that the C-terminal domain of the heptic repeat-2 region of S2 subunit (which plays a role in membrane fusion) has the potential to bind p53, BRCA-1, and BRCA-2 proteins [136]. Should this interaction be confirmed, it would open a dangerous scenario. In fact, the possible sequestration of these proteins by the S2 Spike protein would have catastrophic consequences in the cell because of the loss on the control of genome integrity and cell behavior.

Very recently, Zhang and El-Deiry [137] have tested this hypothesis in various cancer cell lines in which the SARS-CoV2 protein was transgenically expressed. Co-immunoprecipitation did not confirm the interaction between S2 and p53, likely because the two proteins reside in different compartments (cytosol and nucleus, respectively). However, these authors found that the exogenous expression of the Spike protein attenuated the transcriptional activity of p53, and this was not due to the MDM2-mediated degradation of p53 [137]. Of note, when the cancer cells were treated with the DNA-damaging chemotherapeutic drug cis-platinum, the Spike-expressing cells could not transcribe p21 for blocking the cell cycle and to induce cell death.

When the AKT pathway is activated by growth factor receptors, p53 is degraded by the proteasome via MDM2 and this is more likely to occur when ACE2, an alternative substrate of MDM2, is less abundant in the cell. SARS-CoV2 has been shown to trigger the EGFR-AKT pathway and to deplete ACE2 (see Section 5.3 and Section 5.4), a combination that could favor p53 degradation.

### 5.4. Spike Protein Induces Cell–Cell Fusion: A Step Toward Cancer Transformation?

Cell–cell fusion is a well-known phenomenon characteristic of cancer leading to hybrid cells with mixed behavior due to the combined contribution of not only the nuclei but also of cytoplasmatic organelles, among which mitochondria and lysosomes may play a major role [138]. Within the tumor microenvironment, cancer cells may increase their malignant potential by fusing with mesenchymal stem cells, fibroblasts, and macrophages [139]. In addition, the formation of such syncytia within the tumor microenvironment could promote the immune evasion of tumor cells following the entrapment of T and NK cells (forming “cell-in-cell structures”), thus leading to lymphopenia [140]. Cell fusion is relatively rare in spontaneous tumors, yet this event could be promoted by SARS-CoV2 infection. The presence of the polybasic furin-sensitive site enhances the fusogenic property of the S2 subunit of the Spike protein, which can lead to the formation of syncytia [140]. This happens when a SARS-CoV2-infected cell exposes on the membrane the Spike protein that interacts with the ACE2-TMPRSS2 or ACE2-AXL-NRP1 proteins expressed on a neighboring cell [141,142]. This process is believed to enhance cell–cell infectivity.

Worth noting, hydroxychloroquine, an inhibitor of endosomal–lysosomal acidification and of autolysosome formation, was found to inhibit this process [140], which gives credit to the use of this drug for the prophylaxis of COVID-19 [143].

Given that ACE2 and AXL are highly expressed in cancer epithelial cells and in stromal/mesenchymal cells, the ability of Spike protein to promote the fusion of neighboring cells expressing these proteins raises concerns about the possible formation of hybrid tumor cells with increased metastatic potential [144,145].

### 5.5. SARS-CoV2 Replication Dysregulates Autophagy: A Step Toward Carcinogenesis?

Autophagy is a lysosomal-driven degradation process that eliminates damaged and redundant subcellular structures and maintains tissue homeostasis, keeping under check cell proliferation and cell migration [146]. Autophagy is meant to entrap within the autophagosome (a double-membrane vesicle arising from the endoplasmic reticulum) any cytosolic protein agglomerate and organelle that perturbs cell homeostasis and direct their degradation by fusing with lysosomes, acidic organelles endowed with a wide range of hydrolytic enzymes [146]. Perturbation of the autophagy pathway has negative impacts on cell homeostasis and might favor the malignant phenotype [147]. Notably, dysregulated autophagy in cancer cells may instead favor survival against anti-cancer therapies [148]. As we will see, dysregulation of autophagy occurs in SARS-CoV2-infected cells.

Autophagy is involved in coronavirus infection, replication, and viral spreading. SARS-CoV2, like many other viruses, can exploit this vesicular process for its own replication and egression from the cell [149,150]. Particularly, the nonstructural proteins NSP15 and NSP6 can hijack the autophagy pathway so that the former induces the formation of autophagosomes while the latter alters the acidification of the lysosomes impairing their fusion with autophagosomes [149]. In so doing, the virus will escape the lysosomal degradation and instead will divert the nascent autophagosomes versus the formation of double-membrane vesicles for its assembly [35,149]. Another study found that SARS-CoV2 ORF10 localizes at the mitochondria where it binds to mitochondrial antiviral signaling protein and directs its degradation via mitophagy [151]. In SARS-CoV2 infection, autophagy plays an important role in protecting cells from death [152], for instance, it protects from pyroptosis-infected immune cells by degrading the inflammasome [37,38]. Finally, it is worth mentioning that FYCO1 (FYVE and coiled-coil domain autophagy adaptor 1), one of the SARS-CoV2 infection susceptibility genes [56], encodes a RAB7 adaptor involved in autolysosome formation, and it is considered a novel oncogene in that it promotes EMT and migration in breast and cervical cancer cells [153,154].

### 5.6. SARS-CoV2 Alters Mitochondrial Respiration and Induces Oxidative Stress

A link between glucose metabolism and SARS-CoV2 infection emerged with the observation that uncontrolled glycemia was a risk factor for COVID-19 [155]. An important feature of cancer cells is the altered metabolism of glucose known as the Warburg effect by which cancer cells avidly uptake glucose that is fully glycolyzed in the cytosol (to provide substrates for nucleoside synthesis) instead of being completely oxidized via mitochondrial respiration [156]. This divergence in the glucose metabolism is directed by the oncogenic transcription factor HIF-1α (Hypoxia-Inducible Factor-1α), that besides the genes of the glycolytic pathway, also transcribes, among others, genes involved in angiogenesis (VEGF, vascular endothelial growth factor) and cell motility (HGF, hepatocyte growth factor), inflammation, and tumor microenvironment remodeling [157].

It has been reported that SARS-CoV2 induces the glycolytic shift in infected lung macrophages [158]. Mechanistically, the SARS-CoV2 ORF3a induces mitochondrial ROS production that stabilizes HIF-1α, which then promotes glycolysis [159]. A similar glycolytic shift might also occur in SARS-CoV2-infected epithelial cells, and this would be an add-on in the case of pre-neoplastic cells.

To close the circle, IL-6, which drives the cytokine storm in COVID-19, induces the glycolytic shift in cancer cells and promotes the phenoconversion of stromal fibroblasts into permissive cancer-associated fibroblasts through inhibition of autophagy [160,161].

In cancer cells, there is an interplay between the altered glucose metabolism and the mitochondrial respiration [102]. Dysfunctional mitochondria produce oxidative radicals (ROS) that can trigger the inflammasome with production and secretion of inflammatory cytokines. The role of ROS in cancer development and progression depends on how much they are produced: low to moderate levels trigger cell proliferation and migration, high levels of ROS damage the proteins, membranes, and DNA and induce cell death [162]. Notably, ROS may induce or inhibit autophagy (and mitophagy, particularly) with opposite consequences in tumorigenesis and metastasization [163].

SARS-CoV2 infection has been shown to affect mitochondrial respiration. Disruption of mitochondrial morphology and functioning with over production of ROS in peripheral leukocytes and muscles is a common finding in COVID-19 patients [164]. Thus, SARS-CoV2 infection of pre-malignant or dormant tumor cells could elicit such effects triggering tumor growth and invasion.

### 5.7. SARS-CoV2 Triggers the Inflammatory Cytokine Storm and Induces Immune Cell Depletion Leading to a Microenvironment Permissive for Relapses and Metastasis

SARS-CoV2-infected monocytes and macrophages isolated from the lungs of COVID-19 patients produce huge amounts of pro-inflammatory cytokines such as IL-1β, TNF-α, IL-6, and IFN-α, β, and λ, which precedes the so-called cytokine storm and meanwhile abates the T-cell immune response [158]. This production of cytokines was directed by HIF-1α in response to mitochondrial ROS and could, in fact, be prevented by antioxidants such as N-Acetyl Cysteine [158]. Of note, the secretion of SARS-CoV2-infected monocytes, and particularly IL-1β, inhibited the proliferation of CD4+ and CD8+ T cells and increased the surface expression of PD-1 in CD4 lymphocytes, indicative of immune cell exhaustion [158]. These findings are consistent with the reported association between the cytokine storm (particularly IFN-γ and TNF-α) and lymphopenia [165,166]. In infected patients, hyper-production of proinflammatory cytokines (particularly, IL-6, TNF-α, and IFN-γ) may follow the accumulation of undegraded angiotensin II and overactivation of its type 1 receptor (AT1R) because of Spike-induced down-regulation of ACE2 [167].

Pro-inflammatory cytokines recruit myeloid-derived suppressor cells that create a microenvironment permissive for tumor growth and development of metastases by inhibiting the T-cell anti-tumor immune response [168].

Further concern is that the inflammatory response and immune system dysregulation associated with SARS-CoV2 could create the conditions for the awakening of dormant tumor cells [169,170]. Intriguingly, AXL, the receptor for Spike SARS-CoV2 highly expressed in cancer cells, has been shown to be essential for TGF-β2-induced dormancy of metastatic cancer cells [171]. Adding to the complexity, the GAS6-AXL axis induces autophagy in macrophages, inhibiting the activation of the inflammasome and the release of IL-1β and IL-18, thus mitigating inflammation [172]. IL-6, the main culprit of cytokine storm [173], is a pro-tumorigenic cytokine as it promotes cancer cell proliferation and migration, interrupts cancer cell dormancy, and worsens the prognosis of cancer patients through inhibition of autophagy [98,174].

**Figure 2:**
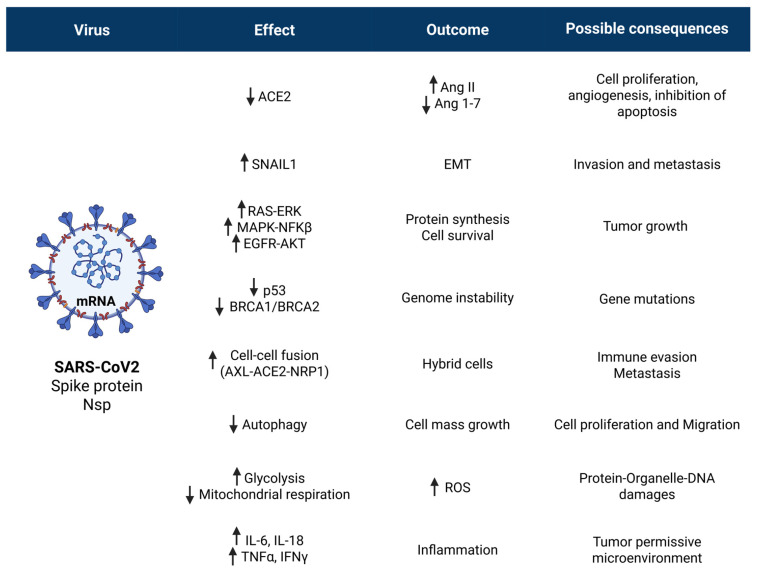
SARS-CoV2 infection can elicit pro-tumorigenic effects by affecting cell and stromal homeostasis. In the cell, spike and other viral proteins may cause ACE2 depletion, trigger cell survival and proliferation pathways, inhibit autophagy, promote cell–cell fusion, and alter metabolism with ROS production, while in the stroma, the viral infection may eventually lead to an inflammatory and immune-depleted tumor-permissive microenvironment. For details, see the text. Arrows up indicate “induction, up-regulation, stimulation“; arrows down indicate “depletion, down-regulation, inhibition”.

It is unknown whether these scenarios can occur in the tumor context of patients infected with the virus, an aspect that deserves to be investigated in depth. The observation that the risk of cancer-related mortality and lung metastasis is higher in SARS-CoV-2-infected patients and the demonstration that IL-6 associated with SARS-CoV2 infection can awaken dormant breast carcinoma cells metastasized in the lung of mice support this possibility [175]. The cellular and systemic effects and possible cancer-related consequences of SARS-CoV2 infection are schematically illustrated in Figure 2.

## 6. The Anti-COVID-19 mRNA Pro-Vaccine and the Cancer

Both the anti-COVID-19 mRNA pro-vaccines BNT162b2 and mRNA-1273 consist of lipid nanoparticles containing the full length mRNA (of 4284 and 4004 nucleotides, respectively) encoding the Spike protein; however, they differ in the 5′ and 3′ untranslated regions, the total amount of mRNA (30 µg/0.3 mL and 100 µg/0.5 mL, respectively), and the chemical composition of the lipid nanoparticles (the cationic ionizable lipid is ALC-0315 and SM102, respectively) [176]. To prevent the prompt degradation of the mRNA pro-vaccine within the transfected cells, all the uridines have been replaced by N1-methyl-pseudouridine, and several codons have been modified in their third nucleoside to optimize translation efficiency [176]. The vaccine Spike is identical to the viral protein (it has the furin-sensitive cleavage site for splitting into the S1 and S2 subunits) except for the substitutions of the amino acids 986 K and 987 V with two prolines to fix the protein in the pre-fusion form [176]. Though stabilized in the prefusion conformation, the vaccine Spike can bind to ACE2 and be cleaved by furin [177]. The above modifications may explain why the mRNA and the intact protein or fragments of the vaccinal Spike persist in the circulation of the vaccine for long time and can be found in organs distant from the injection (deltoid) site [178,179,180,181]. Additionally, the vaccinal mRNA and Spike may travel throughout the body with the exosomes [182], which increases the risk of triggering epiphenomenal reactions associated with severe adverse effects in various organs [108,183,184]. The mRNA vaccine technology relies on the endogenous synthesis of the immunogen (in this case, the Spike protein) that is further processed by antigen processing cells to instruct the lymphocytes for producing neutralizing antibodies and to mount a T-cell immune response [185].

The facts that the exogenous protein driven by a modified mRNA is synthesized within the host cells and that portions of it or of its fragments (since it can be cleaved by furin) can be exposed on the membrane of any cell (since the mRNA is delivered via lipid nanoparticles) increase the risk of deceiving the immune system, which adds on the tissue damages brought by the Spike-ACE2 interaction. The serious and sometimes (fortunately rare) fatal adverse effects associated with mRNA COVID-19 vaccination have been covered in other articles [108,183,184,186,187] and are not the subject of this article as here we focus on the potential pro-carcinogenic effect of the COVID-19 vaccination with such products.

There are several mechanisms and pathways that could link the mRNA anti-COVID-19 vaccination with an increased risk of cancer progression, some of which are common with those connected with SARS-CoV2 infection (the Spike; the inflammatory cytokines) and others that are unique to the mRNA pro-vaccine being associated with its peculiar composition (the presence of pseudouridine; the presence of impurities such as truncated mRNAs and traces of DNA; the presence of inflammatory cationic lipids) and with the vaccination schedule that comprises several shots in a too-short time. The latter has implications as it exposes the vaccinated person to a greater risk of infection, thus facilitating exposure to the side effects of SARS-CoV2 described above.

Unlike the viral Spike protein, the vaccine spike protein has not been associated with dysregulation of autophagy and energy metabolism. However, other factors may link the vaccine Spike protein to processes that potentially increase the risk of carcinogenesis as we will illustrate in the next paragraphs.

### 6.1. The Vaccinal Spike Displays Pro-Carcinogenic Properties Like Viral Spike

The vaccinal Spike shares very similar structural characteristics with viral Spike in terms of binding to surface receptors and thus triggering similar pathways.

In brief, recalling what has been documented for the viral counterpart, vaccinal Spike has the potential to (i) deplete membrane-bound and soluble ACE2 (see Section 5.1); (ii) trigger the oncogenic ERK/MAPK, EGFR-AKT, AXL, and SNAIL-TGFβ pathways (see Section 5.1 and Section 5.2); (iii) interact with ERs in breast cancer cells (see Section 5.2), (iv) interfere with tumor suppressor TP53 stability and transcriptional activity (see Section 5.3); (v) induce the formation of syncytia (see Section 5.4). The consequent effects of these actions include the promotion of cell proliferation and cell migration, induction of EMT, and inhibition of cell death, as discussed in detail in Section 5.

Importantly, the brief protection induced by mRNA pro-vaccines against COVID-19 requires frequent and closely spaced vaccinations, resulting in a tolerogenic immune response and subsequent increased susceptibility to SARS-CoV-2 infection, which creates the conditions under which these non-genotoxic pro-carcinogenic pathways are likely to be activated.

### 6.2. Molecular, Biochemical, Genetic, and Epigenetic Effects of the mRNA Pro-Vaccine: Hypothesizing the Unpredictable

Due to patent protection, data on the manufacturing technology and quality control of COVID-19 mRNA pro-vaccines are scarce [176]. What follows is therefore inevitably theoretical and based on the limited information available.

T7 RNA polymerase-directed in vitro transcription of a DNA template yields the desired RNA, but it also has some drawbacks such as the generation of unwanted RNA species, including double-strand RNA and a mixture of short abortive transcripts of various length. The presence in the market of different batches of vaccine with different composition due to non-standardized manufacturing and quality control remains controversial, denied by some studies and confirmed by other studies [176,188]. In this regard, some batches of BNT162b2 were found to contain on average only 50% of intact Spike-coding mRNA, the rest being fragments of various length [188,189]. These fragments could theoretically impair the synthesis of target proteins functioning as sponges for a variety of cellular mRNAs. However, the sequence of these fragments has never been disclosed, and therefore, their possible interference on the translation of cellular mRNAs remains speculative.

The replacement of uridines with N1-methylpseudouridine deceives the reading machinery in the translation of the mRNA into protein, causing frameshifts in the reading of the codons with the consequence of synthesizing unintended proteins [190] that might have unpredicted consequences [191]. Quite reassuring, a synthetic mRNA resembling the BNT162b2 Spike-coding mRNA with 100% N1-methylpseudouridine was found to translate into intact Spike protein when expressed in HEK293 cells [192]. In vivo, the story goes differently. N1-methylpseudouridine containing mRNAs does not efficiently stimulate dendritic cells, with reduced production of type I Interferon (which exerts anti-cancer functions) and decreased T cytotoxic activity, and this may be relevant for the antitumor immune response [193]. Consistently, BNT162b2 vaccination modulated the innate immune responses by increasing the production of inflammatory cytokines IL-1β and IL-6, while decreasing that of IFN-α [194]. Such immune-suppressive and tumor-permissive scenario was reported in an in vivo (OVA-expressing) melanoma model of cancer vaccination with N1-methylpseudouridine modified mRNA encoding the transgenic tumor antigen (OVA, ovalbumin) encapsulated in lipid nanoparticles [195]. While the unmodified mRNA OVA vaccine elicited antitumor effects characterized by robust infiltration of CD40+ DCs and OVA-specific IFN-γ secreting T cells, the vaccination with pseudo-uridine modified mRNA greatly decreased immunogenicity (decreased IFN-γ production and TNFα-producing CD8+ T cells) in spite of the highest translation efficiency, resulting in increased tumor growth and number of lung metastases [195].

RNA adenosine-to-inosine editing is a co-transcriptional process catalyzed by adenosine deaminase ADAR1 acting on double-stranded portions of immature RNA and potentially resulting in transcriptome and proteome changes, as inosine is read as guanosine. RNA editing may involve coding and non-coding regions and can affect stability, alternative splicing, and translation of mRNAs as well as the processing and specific targeting of non-coding RNAs. Up-regulation of ADAR1 expression and overall increases in RNA editing have been associated with the malignant phenotype [196]. A recent study showed that the expression of ADAR1 in the blood of vaccinees increases with the number anti-COVID-19 mRNA pro-vaccine doses [197]. While this observation is not sufficient to establish a possible cancer link, it is somewhat concerning that among the top three genes with significant A-to-I editing is Slingshot protein phosphatase (SSH), a cofilin phosphatase known to promote cancer invasiveness and metastasis [198].

Another concerning issue for the COVID mRNA pro-vaccine is that the optimization with enriched guanosine–cytosine (GC) and N1-methylpseudouridine may favor the formation of tetrads of guanine called G4 (G quadruplex) that are known to destabilize DNA and are frequently found in cancer [199,200]. G quadruplex is a preferential target of the Polycomb Repressor Complex II, that exerts epigenetic control of gene transcription [200]. However, to elicit a possible damaging effect on the transcription and DNA repair machineries, the G-rich mRNA (fragments) of the pro-vaccine should relocate in the nucleus of the cell. This eventuality seems very unlikely, although it cannot be excluded.

There is however another issue: following a modification in the manufacturing procedure (that now makes use of DNA plasmid instead of PCR to produce Spike mRNA), trace DNA impurities have been found in the BNT162b2 mRNA pro-vaccine [201], although the biological significance of this finding is still unknown and deserves further investigation.

### 6.3. Disruption of the Immune Surveillance and Induction of Inflammation: Creating the Condition for Awakening the Dormant Tumor

In Section 5.7, we have discussed the molecular and cellular mechanisms through which SARS-CoV2 could interrupt tumor dormancy. With respect to SARS-CoV2 infection, the injection with LNP-mRNA pro-vaccine brings additional stress to the tumor microenvironment for the following reasons: 1. Repetitive vaccination shifts the immunogenic response toward a tolerogenic and pro-inflammatory response and overall suppression of the immune response; 2. The lipid component of the nanoparticle is strongly inflammatory.

Due to short term of protection by the anti-COVID-19 mRNA pro-vaccine, repeat vaccination has been recommended on average every 6 months. However, repetitive boosters with Spike mRNA pro-vaccines modulate the adaptive immune system, leading to a shift from an immune to a tolerogenic response. After three doses, a class switch of immune-reactive IgG1 and IgG3 versus the tolerogenic IgG4 was observed in almost half of the vaccinees [63]. A very recent study showed that in children, the serum level of anti-Spike IgG4 continues to raise up to one year after the second dose of Comirnaty [202]. Experiments of vaccination with mRNA coding for the receptor-binding domain of the SARS-CoV2 Spike in mice confirmed that repetitive boosters determine a condition of humoral and cellular immune tolerance [203]. Worth noting, a literature search and meta-analysis found that high levels of IgG4 increased the risk of developing cancer, particularly pancreatic cancer and lymphoma [204]. Local concentration of IgG4, regardless of the antigen specificity, has been shown to drive immune evasion in the tumor microenvironment by inhibiting IgG1-mediated cancer cytotoxicity [205].

T-cell immunity plays a major role in anti-cancer response as well as in keeping dormant (immunogenic dormancy) micrometastases [206]. Unfortunately, multiple vaccinations with anti-COVID-19 mRNA pro-vaccines have been shown to cause T-cell exhaustion and increased expression of PD-1 [86]. A Phase II study reported a transient lymphopenia in some 50% of the vaccinees with one dose (30 or 100 µg) of BNT162b1, and in 33% of those who received the highest dose (100 µg), lymphopenia was of grade 3 [207]. Further contributing to interruption of the dormant-associated immune tumor microenvironment is that these mRNA pro-vaccines can trigger a strong inflammatory response with elevated levels of circulating IL-17 [208] and, particularly in cancer patients previously infected with SARS-CoV2, a shift of memory T-cell toward pro-inflammatory IL-17+ CD8 [84]. IL-17 is known to promote cancer cells’ proliferation in addition to impairing the T-cell mediated anti-tumor response [209].

Increased levels of circulating cytokines (among which IL-6 and IL-17) and growth factors (among which VEGF and bFGF) may be detected in vaccinees up to one year after vaccination with anti-COVID-19 mRNA Comirnaty [210]. Hypothetically, these cytokines and growth factors could interrupt autophagy-mediated [98,174] and angiogenic-mediated [211] tumor dormancy.

Further contributing to an inflammatory potentially tumorigenic microenvironment is the LNP component, which is said to function as an immune stimulator adjuvant. The cationic LNP component of the mRNA pro-vaccine was shown to induce the release of inflammatory cytokines (mainly, IL-6, TNFα and IL-1β) by macrophages and to activate the serum complement via the alternative pathway [212]. This could explain the so-called “radiation recall phenomenon” shown to occur in cancer patients a few days after the second dose of the BNT162b2 mRNA pro-vaccine [213].

**Figure 3:**
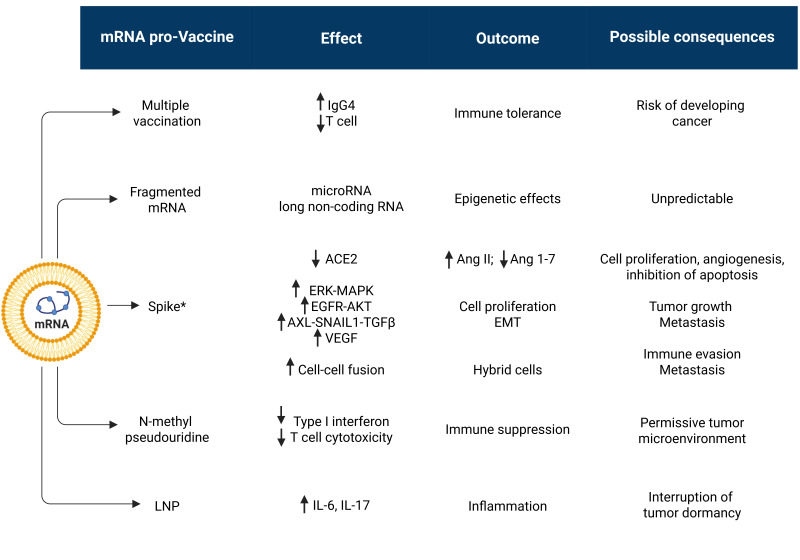
Anti-COVID-19 mRNA vaccinations can elicit pro-tumorigenic effects by affecting cell and stromal homeostasis. Multiple shots, required to maintain immune protection, may eventually lead to an inflammatory and immune-tolerant tumor microenvironment, along with stimulation of angiogenesis, which altogether create conditions for the awakening of dormant tumors. The presence of an intrinsic abnormal mRNA (rich in N-methyl-pseudouridine) and mRNA fragments, along with Spike-mediated effects on membrane receptor signaling, may introduce additional risks that promote malignancy. For details, see the text. (* indicates that Spike protein coded by the mRNA pro-vaccine presents with modified amino acids). Arrows up indicate “induction, up-regulation, stimulation”; arrows down indicate “depletion, down-regulation, inhibition”.

Overall, continued vaccination with these COVID-19 mRNA pro-vaccines impairs the innate and adaptive immune system and sustains an elevated inflammatory state with IL-6 and IL-17 overproduction, along with inhibition of autophagy and stimulation of AXL and VEGFR pathways that altogether are conducive to awakening of dormant tumors and cancer progression. The cellular and systemic effects and possible cancer-related consequences of mRNA anti-COVID-19 vaccination are schematically illustrated in Figure 3.

## 7. Data from the Real World: Case Reports Linking Anti-COVID-19 mRNA Vaccination and Cancer

Although supported by the whelm of data in the literature, the mechanisms and pathways illustrated above are only indicative of the carcinogenic potential of COVID-19 mRNA-based pro-vaccines. There is no specific research focusing on cancer prognosis and fatal events in vaccinated cancer patients in relation to COVID-19, except one study reporting the causal correlation in two out of three patients [71].

How about data from the real world? Recently, a population-based retrospective study in a large cohort of unvaccinated (595,007) and vaccinated (2,380,028) individuals in Seoul (South Korea), where the cumulative incidences and corresponding Hazard Ratio of cancers were measured one year after COVID-19 vaccination, found an association between vaccination and increased risk of thyroid, gastric, colorectal, lung, breast, and prostate cancer [214].

In absence of an active pharmacovigilance to collect data specifically addressing the possible correlation, if not causation, between the COVID-19 mRNA vaccination and cancer, we rely on case reports in the literature.

Table 1 and Table 2 summarize the known cases, respectively referring to the anti-COVID-19 mRNA pro-vaccines from Pfizer and Moderna (Table 1) and to other types of anti-COVID-19 genetic vaccines (Table 2). Although when taken individually the clinical cases can be dismissed as anecdotal facts, when considered altogether, a series of reports converging on the same conclusion should trigger the suspicion and stimulate a discussion in the scientific community.

**Table 1:** Case reports relating cancer to mRNA COVID-19 pro-vaccines.

Disease (Onset)	Clinical Features	Histological–Biological Features	Type of Vaccine	Ref.
Angio-immunoblastic T-lymphoma (onset 6 months after 2nd dose)	66-year-old man presented with lymphadenopathies; increased number, size, and metabolic activity (of lymph nodes 8 days after 3rd dose)	Gene mutations: RHO, TET2, DNMT3A, IDH2	BNT162b2 (March, April, September 2021)	[215]
Recurrence of axillary lymphoproliferative disorder (2 days after 1st dose)	79-year-old man in remission from a primary cutaneous anaplastic large-cell lymphoma cured two years before; presented with ulcerated tumor with surrounding erythema	CD-30-positive lymphoproliferative disorder; TCR gene rearrangement matching the previous 2019 clone	BNT162b2	[216]
Nodal Marginal zone B-cell lymphoma (sudden appearance of temporal mass the day after 1st dose)	80-year-old woman presented with multiple (n. 12) lymphadenopathies at week 6 from 1st dose (week 3 from 2nd dose); increased number (>22) and size (2.5×) in ten weeks	Lymphoid cells positive for CD20, CD79a, and BCL-2; negative for CD3, BCL6	BNT162b2 (2 doses, 3 weeks apart)	[217]
Diffuse large B-cell non-Hodgkin lymphoma (cervical mass appearance one week after 2nd dose)	58-year-old woman presented with tumor mass at the angle of the left parotid gland progressively growing from June to September with multiple reactive lymph nodes, and finally operated in October 2021	Confirmed DLBC NHL positive for CD20, PAX5 and negative for CD30, AE1/AE3; 85% Ki-67 positivity	BNT162b2 22 May; 12 June 2021	[218]
Extranodal malignant non-HodgkinT/NK-cell lymphoma (ulcerative lesions appeared 3 days after 1st dose)	53-year-old man presented (December 2021) with multiple ulcerative oral lesions appeared shortly after the 1st dose which worsened after the 2nd dose	Tumor proliferation with T cells positive for CD3 and CD7, granzyme B, CD30; negative for CD4, CD8, and CD20	BNT162b2 6 November; 28 November 2021	[218]
(A) Acute lymphoblastic leukemia (two days after 1st dose of mRNA vaccine); (B) recurrence of B-Acute lymphoblastic leukemia (after 1st dose of mRNA vaccine); (C) recurrence of acute myeloid leukemia (after the booster with BNT162b2)	(A) 49-year-old woman presenting with petechiae and bicytopenia, diagnosed with B-ALL; (B) 47-year-old woman; two years before diagnosed with B-cell lymphoma in remission in the last 14 months; (C) 67-year-old woman; diagnosed with AML in 2007 and in remission in the last 14 years after bone marrow transplant. She had two doses of inactivated SARS-CoV2 vaccine in July 2021 and mRNA BNT162b2 in September 2021	(A) B-ALL: bone marrow showed 20–30% stained with CD19 diffuse positive TdT in blastic cells; (B) bicytopenia and blasts; (C) 90% blasts	BNT162b2	[219]
Four cases of acute myeloid leukemias, one of which extramedullary	(A) 61-year-old man; 30 days after 3rd mRNA dose; (B) 28-year-old woman; 2 weeks after 2nd dose; (C) 72-year-old man; 5 weeks after the 5th dose; (D) 60-year-old man; 1 month after the 4th dose	(A) 80% blastic infiltration; (B) bicytopenia; blastic infiltration; (C) pancytopenia; 70% blastic infiltration; (D) occipital granulocytic sarcoma of CD34, CD123, and MPO positive immature cells; 30% myeloid blasts	BNT162b2	[220]
Diffuse large B-cell lymphoma (lymphadenopathy was observed one day after the 1st dose)	67-year-old man presented with 6 cm subcutaneous lymphadenopathies mass in the left axilla 2 weeks after the 2nd BNT162b2 vaccination	Large, atypical lymphocytes were positive for CD20, BCL2 and MUM-1/IRF4; negative for CD3; over 80% Ki-67 positivity	BNT162b2 (2 doses)	[221]
Diffuse large B-cell lymphoma (lymphadenopathy was observed two days after the 1st dose)	80-year-old woman presented with enlarged 4.1 cm axillary nodule that developed 1 day after the 2nd dose; two months later the nodule increased to 6 cm and additional lesions appeared in the mesentery and the left cavernous sinus	Germinal center B-cell DLBC lymphoma positive for CD20, BCL6, BCL2; negative for CD3 and MUM-1/IRF4; over 90% Ki-67 positivity	BNT162b2 (2 doses)	[221]
Primary cutaneous anaplastic large cell lymphoma (10 days after the 3rd dose)	76-year-old man presented a fast-growing lesion at the site of the injection 10 days after the 3rd dose. A large erythematous tumor of 6 cm diameter was diagnosed 1 month later. Spontaneous regression after 6 weeks	Anaplastic large cell lymphoma T1bN0M0; positive for CD30, CD4, CD2, CD5, MUM1, and negative for CD20, CD8, TIA1, ALK, EMA, CD56, CD123 and CD68	BNT162b2 (1st and 2nd dose) Moderna mRNA-1273 (3rd dose)	[222]
High-grade sarcoma	73-year-old woman; history of angiomyolipoma in 2019; presented with swelling 2–4 days after 2nd dose developed in 6 cm diameter soft mass in the right upper arm	Grade 3, stage IIIA undifferentiated, pleomorphic high-grade sarcoma	Moderna mRNA-1273 (2 doses)	[223]
Primary cutaneous lymphoproliferative disorders	Series of 14 cases, of which 6 classified as relapse and 8 as primary lesions; complete and partial remission within the 19 months follow-up	N.A.	BNT162b2	[224]
Non-Hodgkin lymphoma (few weeks after the 3rd dose)	66-year-old man presented with right axillary lymphadenopathy developed 10 days after the 3rd dose, which grew up to 7 cm in the following 3 months	Stage-II anaplastic large-cell lymphoma, ALK negative and CD30 positive, over 90% Ki-67 positivity	BNT162b2 (January, February, October 2021)	[225]
Conjunctival classic Kaposi sarcoma (few weeks after vaccine booster)	75-year-old woman with complex ophthalmologic history that includes, among others, uveitic glaucoma OU, epiretinal membrane OU, and cystoid macular degeneration OS, presented with irritated conjunctival area	Conjunctival epithelium shows early squamous metaplasia and positive immunostaining with HHV8 within the CD34 positive vascular proliferation	BNT162b2 (three doses)	[226]
Basaloid carcinoma, wrongly cured as Bell’s palsy for almost 8 months (symptoms appeared 4 days after 1st dose)	56-year-old man; no previous health problems; presented with a massive and aggressively infiltrating basaloid-featured cancer in the right side of his face that rapidly progressed and led the patient to death. CT scan (11 months after vaccination) revealed the presence of infiltrating tumor masses in the parotid gland, likely of cutaneous origin	D-dimer value was 1523 ng/mL (normal range is <500 ng/mL). Biopsy confirmed the diagnosis of basal cell carcinoma	BNT162b2 (one dose)	[227]
Philadelphia-positive B-cell acute lymphoblastic leukemia (five days after the booster vaccination with bi-valent mRNA vaccine)	43-year-old woman; insignificant previous medical history; presented with splenomegaly, severe anemia and thrombocytopenia along with leukocytosis (1.0% neutrophil, 9.0% lymphocyte, 0% monocyte, eosinophil and basophil, and 90.0% blast)	Bone marrow shows 68% blastic infiltration; cells were positive for CD34 and TdT, negative for CD117 and MPO. The p190 BCR-ABL1 gene rearrangement was identified by RT-PCR	Five vaccinations as follows: two doses of Oxford/AstraZeneca (4 June and 31 August 2021); half-dose of Moderna mRNA-1273 (15 January 2022), NovaVax (15 July 2022), and booster dose of the bivalent (Omicron BA.4/BA.5–containing) mRNA-1273 COVID-19 vaccine (January 2023) plus SARS-CoV-2 infection on 19 August 2021	[228]
Epstein–Barr virus-positive marginal zone lymphoma (EBV + MZL) at autopsy (17 days after 1st vaccination)	71-year-old woman with history of methotrexate-treated rheumatoid arthritis; died due thrombosis and multi-organ failure 17 days after vaccination. The autopsy revealed systemic lymphadenopathy comprising atypical lymphocytes and scattered Hodgkin/Reed–Sternberg (H/RS)-like cells	Atypical lymphocytes were positive for CD79a, CD19, EBV-encoded small RNA and MUM-1 and negative for CD3, CD5, CD10, BCL6. H/RS-like cells were positive for CD3	Unspecified the type of anti-COVID-19 vaccine	[229]
Intravascular large B-cell lymphoma at autopsy (105 days after the second dose)	61-year-old woman affected by systemic lupus erythematosus recovered 1 month after vaccination for joint pain, clonic spasms, left-sided paralysis, and fever	Diagnosis of hemophagocytic lymphohistiocytosis with intra- and perivascular infiltration of CD20-positive atypical B lymphocytes in spleen, liver, and lungs	Pfizer BNT162b2 mRNA vaccine (2 doses one month apart)	[230]
Longitudinal melanonychia that progressed into subungual melanoma	53-year-old woman affected by longitudinal melanonychia with no known risk factors for melanoma development	Malignant transformation into acral lentiginous melanoma within 2 years from vaccination	Pfizer BNT162b2 mRNA vaccine (3 doses)	[231]
Breast cancer skin metastasis that manifested 1 month after the 6th dose of mRNA vaccination	85-year-old woman affected by breast cancer that was successfully removed by partial mastectomy with clear margins 2 years before	Metastatic cancer cells in the dermis and epidermis showed pagetoid atypical cells with ample cytoplasm features and were positive for spike protein, but not for nucleocapsid protein of SARS-CoV-2	Pfizer-BioNtech BNT162b2 (six doses in 2 years)	[232]

**Table 2:** Case reports relating cancer to anti-COVID-19 genetic vaccines other than mRNA.

Disease	Clinical Features	Type of Vaccine	Ref.
Pheochromocytoma	63-year-old man; pheochromocytoma (very rare benign tumor) of 7 cm developed few days after the vaccination	Johnson and Johnson COVID-19 vaccine	[233]
Recurrence of cutaneous T-cell lymphoma	T-cell lymphoma has been reported in two patients, who were in remission since many years, after the 2nd	Vaxzevria (Oxford/AstraZeneca)	[234]
EBV-positive, diffuse large B-cell lymphoma	51-year-old man; rapidly growing diffuse large B-cell lymphoma was reported in a heart post-transplanted (under immunosuppressant therapy since many years) 7 days after receiving the 1st dose	ChAdOx1 nCoV-19 vaccine	[235]
Primary cutaneous T-cell lymphoma	28-year-old woman; primary cutaneous T-cell lymphoma (CD31, CD71, CD81 positive) mimicking a panniculitis has been reported in a few days after 1st vaccination	COVID-19 Janssen vaccine	[236]
Chronic myelomonocytic leukemia	74-year-old woman; chronic myelomonocytic leukemia and scleroderma were diagnosed, with first signs manifesting two days after receiving the 1st dose, which then progressed to acute myeloid leukemia, severe anemia, and thrombocytopenia, and eventually died due to COVID-19-associated respiratory failure	Johnson and Johnson COVID-19 vaccine	[237]
Classic Kaposi sarcoma manifested 7 days after the 3rd dose of ChAdOx1 vaccine	73-year-old man with a skin nodule of 2 × 3 × 1 cm HIV negative, positive for CD34 and HHV-8	ChAdOx1 nCoV-19 vaccine	[238]

## 8. Discussion and Concluding Remarks

Anti-COVID-19 vaccination has helped, at least in the early phase of their deployment, to manage COVID-19 by reducing the hospitalizations of the vaccinee and thus relieving the workload of health care workers [239,240], although their real efficacy in protecting from deaths in hospitalized patients has been recently questioned [241,242]. However, the immune protection provided by these mRNA pro-vaccines was found to last for a few months, necessitating additional shots to maintain anti-Spike IgG levels. Vaccines are generally considered safe with respect to potential carcinogenicity, and therefore, their approval normally does not require experimental proof of non-mutagenicity unless the injectable product contains a component never tested in humans and for which it is reasonable to suspect potential mutagenic activity. In the case of the anti-COVID-19 mRNA pro-vaccines, it was deemed that the mRNA coding for the Spike protein and the LNP would not have such mutagenic activity. I am of the same opinion, and personally, I believe that these “vaccines” may not have such activity.

Cancer develops after several decades from exposure to mutagenic substances, yet cancerogenesis might be anticipated in individuals with familial predispositions because of inherited mutations in tumor suppressor genes or DNA repair system genes (see Section 2.1 and Section 5). Thus, even if conducted in animals, the period of observation (generally 6–24 months) would not be sufficient to show the potential carcinogenicity of the vaccines in “healthy” animals (with no cancer predisposing genetic defects) maintained in cages under standard conditions with no extra inflammatory hits.

Still, a series of clinical case reports points to a temporal correlation between vaccination with genetically based anti-COVID-19 vaccines and newly diagnosed cancer and cancer progression.

Cancer eventually emerges clinically after a variety of endogenous, exogenous, and circumstantial events have altered the structure and composition of the parenchyma and the stroma. Besides genetic mutations, epigenetic dysregulation, inflammation (and angiogenesis), immune suppression, dysregulation of autophagy, impairment of DNA damage repair, activation of signaling for proliferation and migration, inhibition of signaling for cell death, increased energetic metabolism, all these events contribute to cancer development and progression, and to the awakening of dormant tumors leading to cancer relapse. A thorough review of the current literature shows that the SARS-CoV2 infection and (multiple) LNP-mRNA vaccinations could elicit a cancer-promoting effect through several mechanisms, including disruption of the immunosurveillance and induction of inflammation in the tumor microenvironment, disruption of autophagy control, disruption of tumor suppressor pathways, and activation of kinase receptors involved in cell proliferation, cell migration, and EMT. A major player in these events is the Spike proteins, which can lead to down-regulation of protective ACE2 and concomitant activation of the AXL pathway.

**Figure 4:**
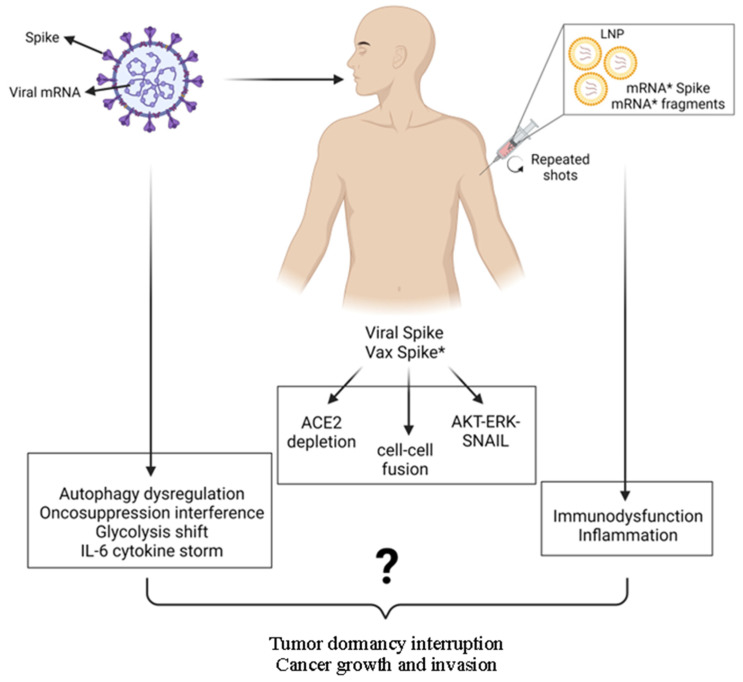
Can SARS-CoV2 infection and repeated mRNA anti-COVID-19 vaccinations be carcinogenic? The SARS-CoV2 virus enters via the aerodigestive tract and exploits the Spike protein to infect only (mainly) the ACE2-positive cells in the lung, intestine, endothelium, and other distant organs including the heart, liver, and kidney. In the infected cell, the virus can be degraded by the autophagy–endocytic–lysosomal pathway or can reproduce and exit to infect neighboring cells. The anti-COVID-19 vaccine is made of liponanoparticles (LNP) containing the modified mRNA coding for the Spike protein, and possibly also fragments of the modified mRNA. The vaccine is injected into the deltoid muscle fibers which will expose on the membrane the S protein to alert the immune system. The S protein and its fragments can be released and be endocytosed by antigen processing cells (macrophages and dendritic cells) to trigger the production by activated B cells. However, the LNP may transfect the vaccine mRNA to any cell. In addition, the vaccine mRNA and the Spike can travel to distant organs within the exosomes. Both SARS-CoV2 and LNP-mRNA anti-COVID-19 vaccine affect cell homeostatic processes and induce immune dysfunction and tissue environment inflammation, all conditions that could potentially lead to the awakening of a dormant tumor or micrometastasis and promote cancer proliferation and invasiveness. In detail, the Spike protein (either viral- or vaccine-derived) can 1. lead to depletion of ACE2, which results in abundance of angiotensinogen 2 having mitogenic and angiogenic properties (while the Ang1-7 produced by ACE2 would have pro-apoptotic and anti-angiogenic properties); 2. cause cell–cell fusion, which results in polyploidy and abnormal chromosomal arrangements; 3. promote the AKT and ERK proliferation pathways and the SNAIL migratory pathway. In addition, the SARS-CoV2 viral infection might interfere with oncosuppressor pathways, disrupt the control of autophagy, induce the glycolytic shift, and trigger the IL-6-driven cytokine storm. On the other hand, repeated cycles of vaccination with the modified mRNA causes immune dysfunction, leading to IgG4-mediated tolerance and disruption of immune surveillance while the LNP causes inflammation. Inflammation (and angiogenesis) and immune suppression created a tissue microenvironment permissive for awakening dormant tumors and micrometastases, thus promoting cancer cell growth and invasiveness. (* indicates that the vaccine mRNA is modified. LNP, liponanoparticle).

These events could combine and be redundantly activated in patients who have been vaccinated and have contracted the infection several times, and in a relatively short time. This unfortunate situation (cocktail effect) would determine a synergism of the damages and alterations caused by the virus and the mRNA pro-vaccine, which can lead to “catastrophic” effects: awakening of dormant tumors (minimal residual disease; micrometastases), and fast progression of cancer (Figure 4). This scenario would be more probable in oncologic patients and in individuals with undiagnosed cancer, and even more in individuals susceptible to cancer because of predisposing genetic defects. An alerting, albeit ignored, signal was reported in a multicenter study where cancer progression and death were reported in some vaccinated patients [71]. As for non-cancer patients, an emblematic case is that of a 43-year-old woman with no significant clinical history who was diagnosed with Ph-positive ALL a few days after vaccination with the double mRNA-1273 vaccine administered in addition to four previous vaccinations with different anti-COVID-19 vaccines plus SARS-CoV2 infections [228].

It is to be noted that the clinical cases discussed here do not establish a causal relationship between the vaccine and the cancer. Such an assessment would require an “ad hoc” investigation [15].

In the practical impossibility to demonstrate a causal link, the biological plausibility of the link between the SARS-CoV2 virus and the anti-COVID-19 mRNA pro-vaccine with cancer must suggest caution in using these types of vaccines and meanwhile adopt appropriate measures to protect the patient at risk (particularly cancer patients) from the infection, while waiting for vaccine developers to take into account what is expressed here to design safer and more effective vaccines. The present observations call for extra caution when using this type of vaccines, taking into consideration the potential risk of triggering the awakening of dormant cancers or of facilitating the development of cancer in individuals with a genetic predisposition to cancer. First and foremost, it is imperative to elucidate the mechanisms underlying the complex interplay between the virus and vaccination, on the one hand, and oncogenic pathways and the immune system, on the other. This knowledge would also help better stratify patients who truly need vaccination and could inform how to prevent and treat unwanted side effects. In other words, it is advisable to perform a personalized assessment of the real need to vaccinate patients at risk, guided by the principles of vaccinomics and adversomics [52,243]. In the meantime, the adoption of other strategies to protect cancer patients, for instance, by using monoclonal antibodies [244] and convalescent hyperimmune plasma [245], is advised.

## Data Availability

Not applicable.
